# Aqueous cinnamon extract ameliorates bowel dysfunction and enteric 5-HT synthesis in IBS rats

**DOI:** 10.3389/fphar.2022.1010484

**Published:** 2023-01-09

**Authors:** Lijuan Yu, Chunhua Huang, Wei Yang, Zhenxing Ren, Lifeng Li, Huiyuan Cheng, Chengyuan Lin, Lixiang Zhai, Ziwan Ning, Hoileong Xavier Wong, Quanbin Han, Wei Jia, Zhaoxiang Bian, Ling Zhao

**Affiliations:** ^1^ School of Chinese Medicine, Hong Kong Baptist University, Hong Kong SAR, China; ^2^ College of Basic Medicine, Guangzhou University of Chinese Medicine, Guangzhou, China; ^3^ Centre for Chinese Herbal Medicine Drug Development Limited, Hong Kong Baptist University, Hong Kong SAR, China; ^4^ Center for Translational Medicine, Shanghai Jiao Tong University Affiliated Sixth People’s Hospital, Shanghai, China; ^5^ Academy of Integrative Medicine, Shanghai University of Traditional Chinese Medicine, Shanghai, China

**Keywords:** cinnamon, irritable bowel syndrome, 5-HT metabolism, tryptophan hydroxylase 1, enterochromaffin cells

## Abstract

Cinnamon protects against irritable bowel syndrome with diarrhea (IBS-D) in humans, but its efficacy and underlying mechanism of action remain poorly understood. Maternally separated (MS) IBS-D rat model and 2,4,6-trinitrobenzene sulfonic acid (TNBS)-induced post-inflammatory IBS-D rat model are characterized by visceral hyperalgesia and diarrhea. This study used the two models to evaluate the effect of cinnamon extract (CE) on bowel symptoms. The MS rat model was also used to explore its underlying anti-IBS mechanism. cinnamon extract reduced defecation frequency and visceral hyperalgesia in MS rats in a dose-dependent manner and effectively improved visceral hyperalgesia in TNBS rats. The efficacy of cinnamon extract was comparable to the positive drug serotonin receptor 3 (5-HT3) selective antagonist, Ramosetron. Excessive 5-HT, a well-known pathogenic factor for IBS, in the colon and circulation of IBS rats was reduced after cinnamon extract intervention. Both, gene and protein levels of the colonic 5-HT synthetase, Tryptophan Hydroxylase 1 (Tph1), were also decreased in CE-treated IBS rats. In addition, a luciferase assay revealed that cinnamon extract and its major components, catechin, procyanidin B1/2, cinnamic acid, and cinnamyl alcohol, significantly inhibited *Tph1* transcription activity *in vitro*. These findings illustrated that aqueous cinnamon extract partially attenuated bowel symptoms in IBS models by directly inhibiting Tph1 expression and controlling 5-HT synthesis. This provides a scientific viewpoint for the use of cinnamon as a folk medication to treat IBS.

## Introduction

Irritable bowel syndrome with diarrhea (IBS-D), a subtype of IBS characterized by abdominal discomfort and frequent defection, is the most prevalent functional gastrointestinal disorder (FGID) both in China and globally (Liu and Liu, 2021; Sperber et al., 2021). Anti-diarrheal medications such as anticholinergics, mu-opioid agonists, and serotonin receptor antagonists are still widely used in IBS-D therapy but have mixed efficacy and can cause side effects (Camilleri and Andresen, 2009). As a result, patients often seek alternative medicines or non-medical approaches to treatment.

Functional foods, defined as foods that offer health benefits beyond their nutritional value, mainly include herbs and spices, which have become increasingly popular in the treatment of irritable bowel syndrome (IBS) because of their advantages of medical values, weak toxic effects, and substantial bowel-improving effects (Hasler, 2002; Sahoo et al., 2014; Fifi et al., 2018). Cinnamon (genus *Cinnamomum*, family Lauraceae) is a herbal spice that is rich in structurally diverse phytochemically active compounds with antioxidant, anti-inflammatory, anti-tumor, anti-diabetic, and anti-microbial properties (Rao and Gan, 2014). When used as a home remedy for IBS, particularly IBS-D, cinnamon can effectively reduce abdominal pain and gut dysmotility (Rao and Gan, 2014). Cinnamon is also served as the key herbal constitute of Traditional Chinese Medicine formulas for treating IBS-D in China (Xiao et al., 2015). However, the anti-IBS effect of cinnamon and its underlying mechanism has not been assessed.

Studies show that high peripheral availability of serotonin (5-HT) contributes to IBS pathophysiology (Camilleri, 2012). Herein, two IBS-D models induced by maternal separation (MS) or 2,4,6-trinitrobenzene sulfonic acid (TNBS), both characterized by IBS-like symptoms and enteric 5-HT overproduction(Qin et al., 2019; Wong et al., 2019), were established to examine the efficacy of CE. The MS model was further used to explore the 5-HT-relevant therapeutic mechanism of CE. Enteric 5-HT was evaluated at the gene, protein, and metabolic levels. The mechanism by which cinnamon affects 5-HT synthesis was also assessed using *in vitro* experiments and reporter gene assays. This study provides a scientific basis for the classic and folk usage of the functional spice, cinnamon, in IBS-D therapy.

## Materials and methods

### Preparation of aqueous CE

Cinnamon bark (*Cinnamomum cassia*) was purchased from the outpatient pharmacy of Hong Kong Baptist University (Hong Kong, China) and identified by professor Quanbin Han from the School of Chinese Medicine at Hong Kong Baptist University. Aqueous CE was prepared by grinding 2 kg of cinnamon into a powder. The powder was soaked in 10x water for 30 min at room temperature, refluxed 2 h in boiling hot water for three times, then vacuum filtrated. The collected solution was concentrated under reduced pressure and further lyophilized using a benchtop freeze dry system (Labconco FreeZone, United States) to create a water extract (the extraction yield was 6.67%). All extracts were stored at -35°C.

### Chemical characterization of cinnamon extract

Using a method previously described by Cheng *et al.*(Cheng et al., 2012), phytochemical analysis of aqueous CEs was conducted with an Agilent 1290 UHPLC system (Agilent Technologies, Santa Clara CA, United States) tandem Agilent 6543 quadrupole time-of-flight mass spectrometer (QTOF-MS, Agilent Technologies, Santa Clara, CA, United States). Chemical components were separated using a 1.7 µm C18 column (2.1 mm × 100 mm, Waters, Milford, MA, United States) with Solvent A (.1% formic acid in water), and Solvent B (.1% formic acid in acetonitrile) at a flow rate of .4 ml/min and a temperature of 40°C. The solvent gradient programming was as follows: 0–10 min, 5%–35% B; 10–18 min, 35%–75% B; 18–21 min, 75%–100% B; 21–24 min, 100% B; 24–24.1 min, 100%–5% B, 24.1–28 min, 5% B. The injection volume was 2 μl. The QTOF-MS was applied in the positive ion mode. The mass scan range was set from 100 to 1700 m/z and the collision energies were set as 15 eV and 35 eV for the target MS/MS acquisition. Ion chromatogram plots containing the dominant chemical components are shown in Supplementary Figure S1.

### Animal experiments

Pregnant Sprague Dawley rats (14–15 days gestation) and adult 8-week-old male Sprague Dawley rats were purchased for IBS modeling from the Laboratory Animal Services Centre at the Chinese University of Hong Kong, China. Rats were housed and maintained at 25°C on a 12 h–12 h alternating light-dark cycle with free access to standard chow diet and drinking water. Animal experiments were performed in accordance with the guidelines from the Committee on the Use of Human and Animal Subjects in Teaching and Research at Hong Kong Baptist University and the protocol was approved (Ref ID: REC/19–20/0163). The MS rat model was established as previously described (Bian et al., 2010). In brief, on postnatal day (PD) 2–14, pups were separated from their mothers and placed into individual cages once a day for 3 h. The pups were returned to their home cage and left undisturbed after the end of each separation while non-handled (NH) pups remained in their home cages as usual. On PD 22, all rat pups were weaned from their mothers and only male pups were used to avoid hormonal cycle-induced variation.

As described in our previous study (Qin et al., 2019), adult male rats were used to establish post-inflammatory IBS model induced by an intracolonic enema with TNBS (P2297, Sigma) solution (5 mg in .8 ml with 50% alcohol). Control mice received a saline infusion. Briefly, after 24 h fasting, a soft plastic catheter was gently inserted into the rat’s colon about 8 cm from the anus. TNBS or saline was instilled into the colon and rats were kept in a head-down vertical position for 5 min to prevent leakage. The whole modeling construction was infused with TNBS or saline 1 time. Thirty days later after TNBS infusion, the abdominal pain threshold of each rat was collected from the abdominal withdrawal reflex (AWR) test and those with visceral hypersensitivity (pain threshold under 40 mmHg) were determined to be post-inflammatory IBS models.

Three doses of aqueous CE extracts (30, 90, and 270 mg/kg/d), previously shown to be effective in animals and humans (Kwon et al., 2011; Hajimonfarednejad et al., 2019), were orally administered daily to either adult MS or TNBS model rats. The 5-hydroxytryptamine receptor 3 (5-HT_3_) selective antagonist, Ramosetron (.03 mg/kg/d, HY-B0595, MCE), was orally gavaged as a positive control as described previously (Asano et al., 2017). Intervention of CE or Ramosetron lasted 2 weeks.

### Assessment of bowel phenotypes

Fecal consistency, defecation frequency, and visceral sensitivity were measured according to our published protocol (Zhao et al., 2018). Feces were collected within 2 h, weighed, and dried. Fecal water content (%) was calculated as it follows (%)= (wet weight - dry weight)/wet weight × 100%. Fecal consistency was included with the fecal water content. Defecation frequency was determined by the accumulation of fecal pellets within 2 h. The AWR test was separately conducted on MS rats on postnatal days 55 and 70. For the TNBS model, 30 days after the enema, the AWR test was separately done before and after CE or Ramosetron intervention on day 31 and day 45. As previously described (Al-Chaer et al., 2000; Bian et al., 2010), rats were anesthetized with ether inhalation and a 6 cm-long flexible latex balloon was inserted into the descending colon. Colorectal distension was initiated with increases of 5 mmHg until a visible contraction of the abdominal wall was observed. The threshold of colorectal distension was recorded and repeated five times.

### Semi-quantification of neuroactive metabolites

Neuroactive metabolites including histamine, glutamate, norepinephrine, acetylcholine, *γ*-amino butyric acid (GABA), dopamine, tryptophan (Trp), 5-HT, and 5-hydroxyindoleacetic acid (5-HIAA) that are closely related to IBS pathogeneses were semi-quantified in rat serum and colonic tissues using ultra-performance liquid chromatography-tandem mass spectrometry (UPLC-MS/MS). All chemical standards were purchased from Sigma-Aldrich (St. Louis, MO, United States). HPLC grade organic reagents for mass spectrometry were purchased from Sigma-Aldrich (St. Louis, MO, United States). UPLC-MS/MS (Agilent UHPLC 1290, United States; Agilent QQQ-MS 6460, United States) was used to analyze the metabolites. Using a single 10-min acquisition with positive ion switching, metabolites were semi-quantified in the multiple reaction monitoring (MRM) mode and separated using an ACQUITY BEH C18 column (1.7 μm, 2.1 × 50 mm) with a linear gradient of .1% formic acid (FA) in water A) and .1% FA in acetonitrile B). The flow rate and injection volume were adjusted to .4 ml/min and 1 μl per sample, respectively. The gradient program was set as follows: 0 min 2% B, 4 min 30% B, 6 min 100% B, 8 min 100% B, 8.1 min 2% B, and 10 min stop. In the positive modes, the column temperature and capillary voltage of the mass spectrometer were set at 40°C and 3.5 kV. The peak integration, calibration equations, and quantification of individual metabolites in the acquisition data were analyzed using Agilent Mass Hunter Workstation Software. The specific MRM transitions and MS/MS parameters of the tested metabolites included the following: histamine (112.1–95 [M + H]^+^), glutamate (148.1–84 [M + H]^+^), norepinephrine (170.1–152 [M + H]^+^), acetylcholine (146.1–87 [M + H]^+^), GABA (104.1–69 [M + H]^+^), dopamine (154.1–137 [M + H]^+^), Trp (205.1–188 [M + H]^+^), 5-HT (177.3–160 [M + H]^+^) and 5-HIAA (192.1–146 [M + H]^+^).

### Quantitative PCR analysis

Total RNA was extracted from cells or tissues using TRIzol reagent (Invitrogen) according to the manufacturer’s instructions. RNA from each sample was reverse transcribed into cDNA using PrimeScript RT master mix (RR036A, Takara). Finally, cDNA templates were amplified with specific primers and 5x SYBR Green PCR Master Mix (RR420A, TAKARA) using the ABI ViiA seven real-time PCR system (Applied Biosystems, United States). The fold change of target gene expression was normalized to *ß*-Actin and presented as 2^−ΔΔCt^ using the comparative Ct method. Primer sequences for qPCR analyses are summarized in Supplementary Table S1.

### Western blot analysis

Protein was extracted from the colonic tissues using radioimmunoprecipitation assay buffer, and then separated by 8% SDS-PAGE and electrophoretically transferred to a PVDF membrane (Millipore, IPVH00010). The membrane was blocked with 5% milk for 1 h at room temperature and incubated with primary anti-TPH antibody (1:1000, Invitrogen, PA1777) overnight at 4°C. After washing with PBST, the membranes were incubated with appropriate secondary antibodies for 1 h at room temperature. X-ray film (Fuji) and an ECL kit (Thermo Fisher Scientific, United States) were used to detect the positive immunoreactions. The relative expression of proteins was quantified using ImageJ software (ImageJ. NIH, United States).

### Immunohistochemical analysis

Paraffin-embedded tissue sections were de-paraffinized and rehydrated with graded ethanol. PBS containing 3% H_2_O_2_ was used to block the endogenous peroxidase activity of the samples. The slides (5 μm thick) were incubated with rabbit anti-Chromogranin A (ChgA) polyclonal antibody (1:200, Abcam, ab45179) overnight at 4°C and probed with secondary antibodies (1:500, Invitrogen) for 60 min. Peroxidase activity was visualized using a Dako LSAB + System-HRP Kit according to the manufacturer’s instructions (Dako Cytomation). Images were captured using a manual inverted microscope Leica DMI3000 B. The ChgA immunostaining densities from five random fields were analyzed.

### Cell cultures and cell viability assay

Human embryonic kidney 293 (HEK293) cells were purchased from American Type Culture Collection (ATCC; Rockville, MD, United States). HEK293 cells were cultured in complete high glucose Dulbecco’s Modified Eagle Medium (DMEM) medium which contained 10% Fetal bovine serum (FBS) and 1% penicillin/streptomycin (P/S, GIBCO, United States) at 37°C in a humidified atmosphere of 5% CO_2_. FBS and DMEM were purchased from HyClone (Logan UT, United States). The 3-(4,5-dimethylthiazol-2-yl)-2,5-diphenyl-2H-tetrazolium bromide (MTT) assay was performed to detect the impact of CE on the cell viability of HEK293 cells. HEK293 cells (3 × 10^3^/well) in complete DMEM medium were seeded in 96-well plates. After 24 h seeding, the supernatant was removed and cells were treated with concentrations of CE (31.25, 62.5, 125, 250, 500, 1000, 2000, and 4000 μg/ml) for 24 h, such dose range setting was referred to the *in vitro* effective doses of CE previously reported (Lin et al., 2008; Tung et al., 2008; Kwon et al., 2009). MTT (20 μl; 5 mg/ml) was then added to each well and the microplate was incubated at 37°C in 5% CO2 for 4 h. Following incubation, the supernatant was removed from each well and 150 μL DMSO was added to dissolve the purple formazan crystals. Optical Density (OD) value of each well was measured at the wavelength of 570 nm using a microplate reader (SpectraMax iD5, Molecular Devices).

### Analysis of *Tph1* transcriptional activity

The Tph1-promoter plasmid was constructed using the nucleotide sequence from positions -2000 to + 32 of the Tph1 gene inserted into a pGL3-basic plasmid which expresses firefly luciferase. HEK293 cells were seeded into 96-well plates and transiently co-transfected with the Tph1-promoter plasmid or a Renilla luciferase-expressing plasmid as a control (total DNA 500 ng/well; 1:1) using Xfect (631,317, TAKARA). After a 4 h transfection, the cells were treated with CE (31.25, 62.5, 125, 250, 500, 1000, 2000 and 4000 μg/ml) or its major components (catechin (HY-N0898, MCE), procyanidin B1(HY-N0795, MCE), procyanidin B2 (HY-N0796, MCE), cinnamic acid(HY-N0610A, MCE), cinnamyl alcohol (HY-Y0078, MCE) and cinnamic aldehyde (HY-N0609, MCE)) at the concentration of 50 μM for 24 h. Such dose of components was set referring to previous *in vitro* works (Ho et al., 2013; Lv et al., 2017). The cells were then lysed by adding 100 μl passive lysis buffer (Promega, United States) to each well, and luciferase activity was detected using the Dual-Luciferase Assay Kit (Promega, United States). Relative luciferase activity of the Tph1-dependent firefly luciferase was normalized to that of Renilla luciferase. All values are expressed as the firefly/Renilla ratio.

### Statistical analysis

Data are presented as the mean ± S.E.M. Statistical analysis was conducted using SPSS 12.0 software. Differences between two groups were determined using a Student’s t-test. When multiple groups were compared, data were analyzed using a one-way ANOVA followed by a Student-Newman-Keuls (SNK) test. Differences were considered significant when *p* < .05.

## Results

### Cinnamon extracts ameliorate bowel symptoms in IBS rats

**FIGURE 1:**
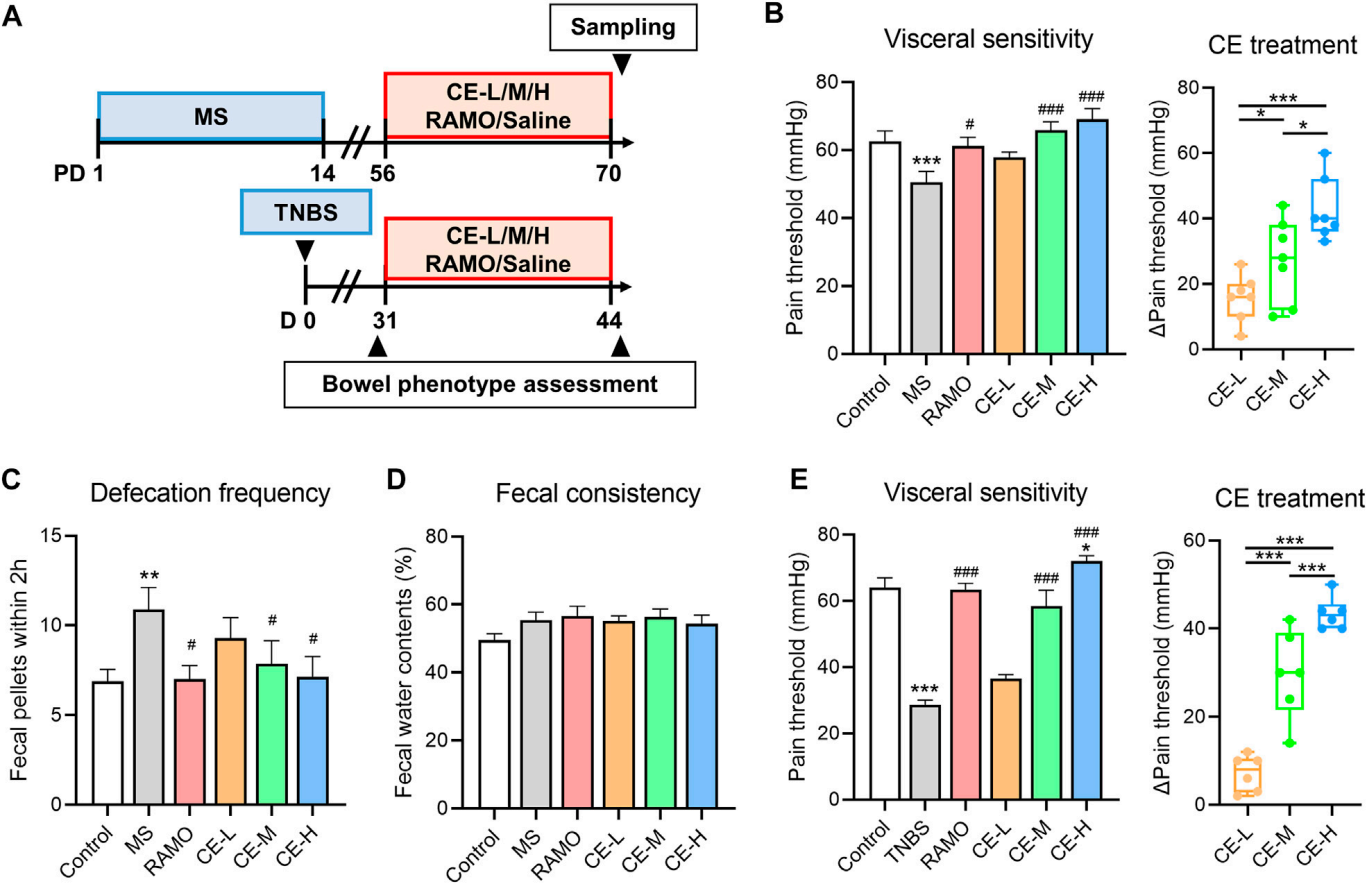
Cinnamon extract (CE) ameliorates bowel symptoms in maternally separated (MS) or 2,4,6-trinitrobenzene sulfonic acid (TNBS)-induced IBS rats **(A)** Timeline of drug interventions and assessments in rat models. MS rats (n = 7–8/group) were individually treated with saline, Ramosetron (.03 mg/kg/d), low (30 mg/kg/d), medium (90 mg/kg/d), or high doses (270 mg/kg/d) of CE for 14 consecutive days while rats without MS handling (n = 8) were used as controls **(B–D)** Bowel phenotypes, including visceral sensitivity, defecation frequency, and fecal consistency of MS rats receiving different interventions **(E)** Visceral sensitivity of control and TNBS rats receiving different interventions (n = 6–8/group). One way ANOVA analysis was used for statistics and significance was expressed by *, *p* < .05; **, *p* < .01; ***, *p* < .005 compared with the control group or within CE groups; #, *p* < .05; ##, *p* < .01; ###, *p* < .005 compared with the model group.

IBS model rats were administrated different doses of CE, Ramosetron, or saline for 14 consecutive days (Figure 1A). Bowel symptoms, including defecation frequency, visceral sensitivity, and fecal consistency were assessed. Defecation frequency and fecal water content were significantly increased in MS models, along with a reduction in visceral pain threshold, indicating that IBS-like bowel symptoms were present (Figures 1B–D). Treatment with Ramosetron or either a medium or high dose of CE reduced all bowel symptoms except for fecal consistency. Of note, CE reduced visceral hyperalgesia in a dose-dependent manner. The dose-dependent analgesic effect of CE was confirmed using another TNBS-induced IBS rat model that primarily exhibits visceral hyperalgesia and intestinal inflammation (Figure 1E). There were no differences in defecation frequency and fecal water content among the TNBS rats (data no shown). These findings highlighted the substantial therapeutic effect of CE on IBS-like bowel symptoms, including a specific dose-dependent amelioration of visceral hyperalgesia.

### Cinnamon extracts decrease circulating and enteric 5-HT bioavailability in IBS rats

**FIGURE 2:**
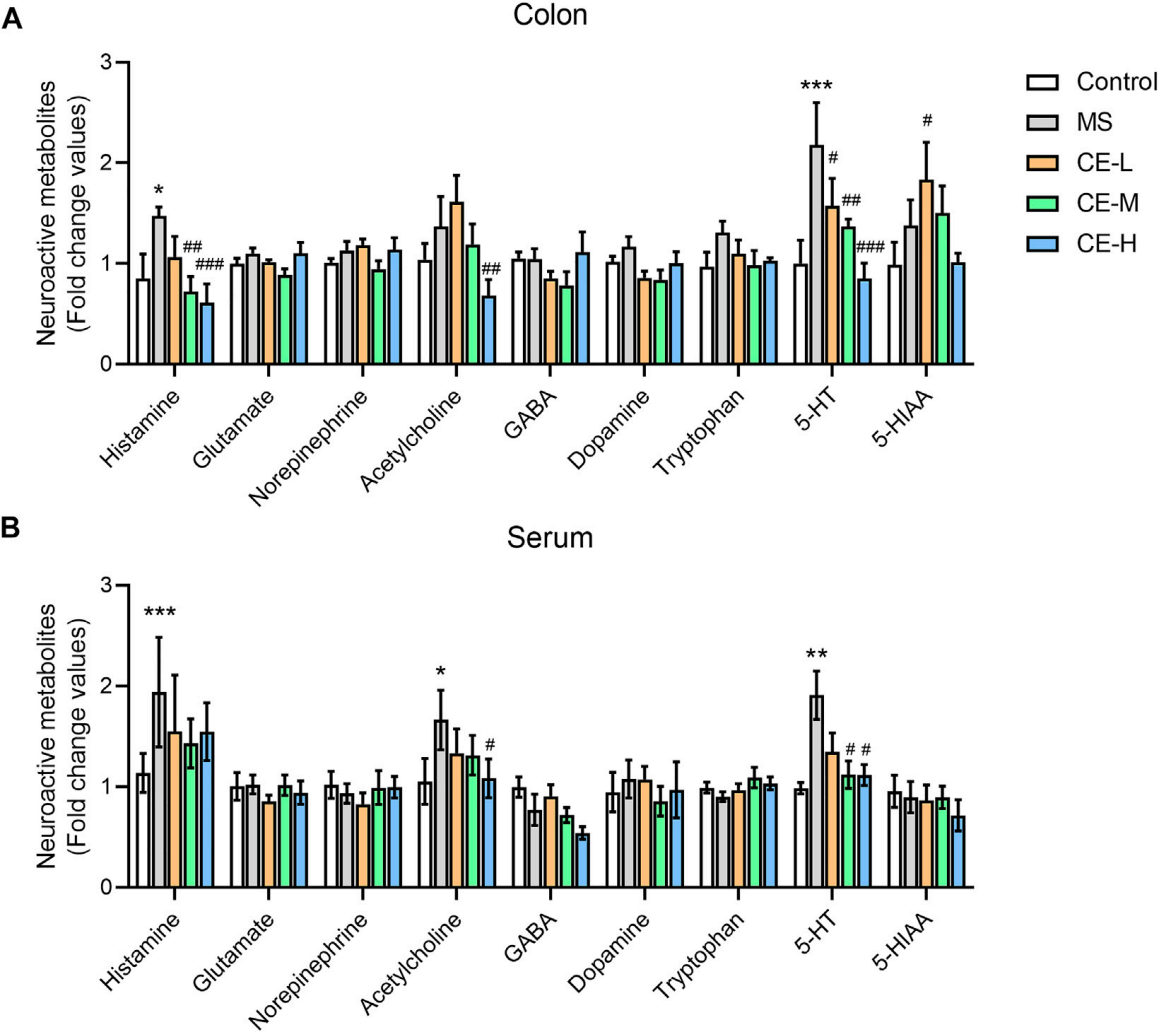
CE decreases circulating and enteric 5-HT bioavailability in MS rats (n = 6–8/group). Neuroactive metabolite levels in rat colonic tissues **(A)** and serum **(B)** were semi-quantified using UPLC/TQ-MS. One way ANOVA analysis was used for statistics and significance was expressed as *, *p* < .05; **, *p* < .01; ***, *p* < .005 compared with the control group; #, *p* < .05; ##, *p* < .01; ###, *p* < .005 compared with the MS model group.

Neurotransmitter dysfunction that impacts motor and sensory activity in the colon is thought to contribute to IBS pathogenicity (Lacy, 2016; Gros et al., 2021). Neurotransmitters and derivates were quantified in rat serum and colonic tissues to explore the underlying therapeutic mechanism of CE using a UPLC-MS/MS-based metabolomic platform. MS-induced IBS rats were found to have higher levels of histamine and 5-HT in serum and colonic tissues than control rats. CE had a suppressive impact on colon histamine and 5-HT levels in a dose-dependent manner (Figure 2A). Moreover, both medium (90 mg/kg/d) and high doses (270 mg/kg/d) of CE significantly reduced serum 5-HT in model rats but had no impact on histamine (Figure 2B). These findings demonstrated that CE has an inhibitory effect on circulating and colonic 5-HT bioavailability in IBS rats.

### Cinnamon extracts downregulate colonic 5-HT synthesis and Tph1 expression in IBS rats

**FIGURE 3:**
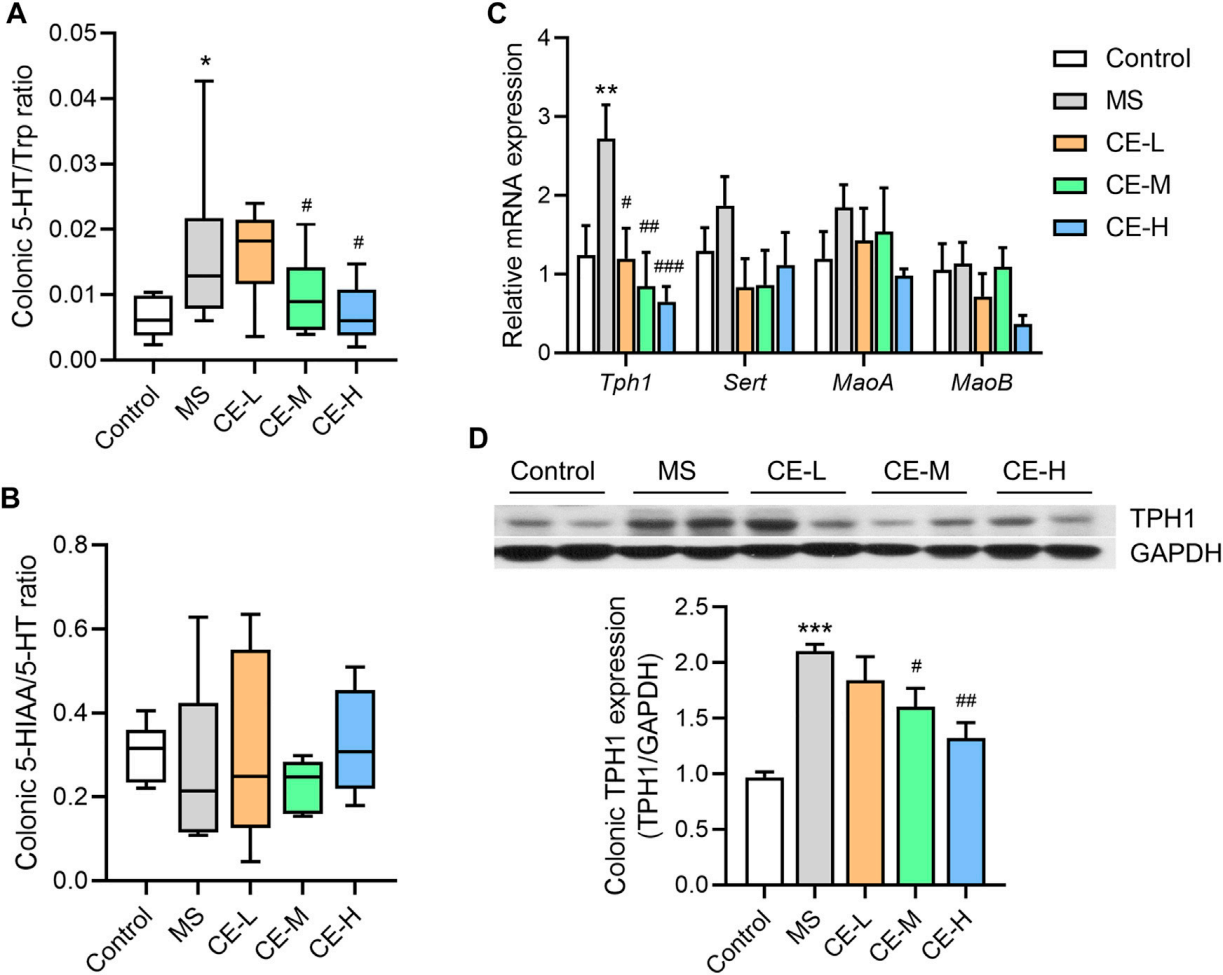
CE inhibits colonic 5-HT synthesis and TPH1 protein expression in MS rats **(A,B)** The ratios of 5-HT to Trp and 5-HIAA to 5-HT (n = 5–6/group); **(C)** Relative expression of colonic genes related to 5-HT synthesis and catabolism (n = 6/group); **(D)** Expression of colonic TPH1 protein among different rat groups (n = 3/group). One way ANOVA analysis is used for statistics and significance is expressed as *, *p* < .05; **, *p* < .01; ***, *p* < .005 compared with the control group; #, *p* < .05; ##, *p* < .01; ###, *p* < .005 compared with the MS model group.

Studies indicate that 5-HT is synthesized from Trp by the rate-limiting enzyme, tryptophan hydroxylase (TPH1), transported into enterocytes *via* the serotonin transporter (SERT), and subsequently catabolized into 5-HIAA *via* monoamine oxidase (MAO) (Visser et al., 2011). Thus, these metabolites and proteins were examined to elucidate the possible mechanism by which CE regulates 5-HT levels. Both medium and high doses of CE were able to reduce the ratio of 5-HT to its synthetic precursor, Trp, in the colonic tissues of MS model rats (Figure 3A). However, there was no difference in the ratio of the catabolic product, 5-HIAA, to 5-HT among the treatment groups (Figure 3B). QPCR analysis also found that CE was able to ameliorate the increase in 5-HT synthetase, Tph1, gene expression in the MS group (Figure 3C). Notably, the inhibitory impact of CE on Tph1 mRNA and protein expression was dose-dependent (Figure 3D). At the same time, there was no difference in the expression of other genes, *Sert* and *MaoA/B*, among the groups. These results demonstrate that CE is likely able to reduce colonic 5-HT levels in MS rats by targeting 5-HT synthesis.

### Cinnamon extracts have no significant impact on colonic enterochromaffin cell hyperplasia in MS models

**FIGURE 4:**
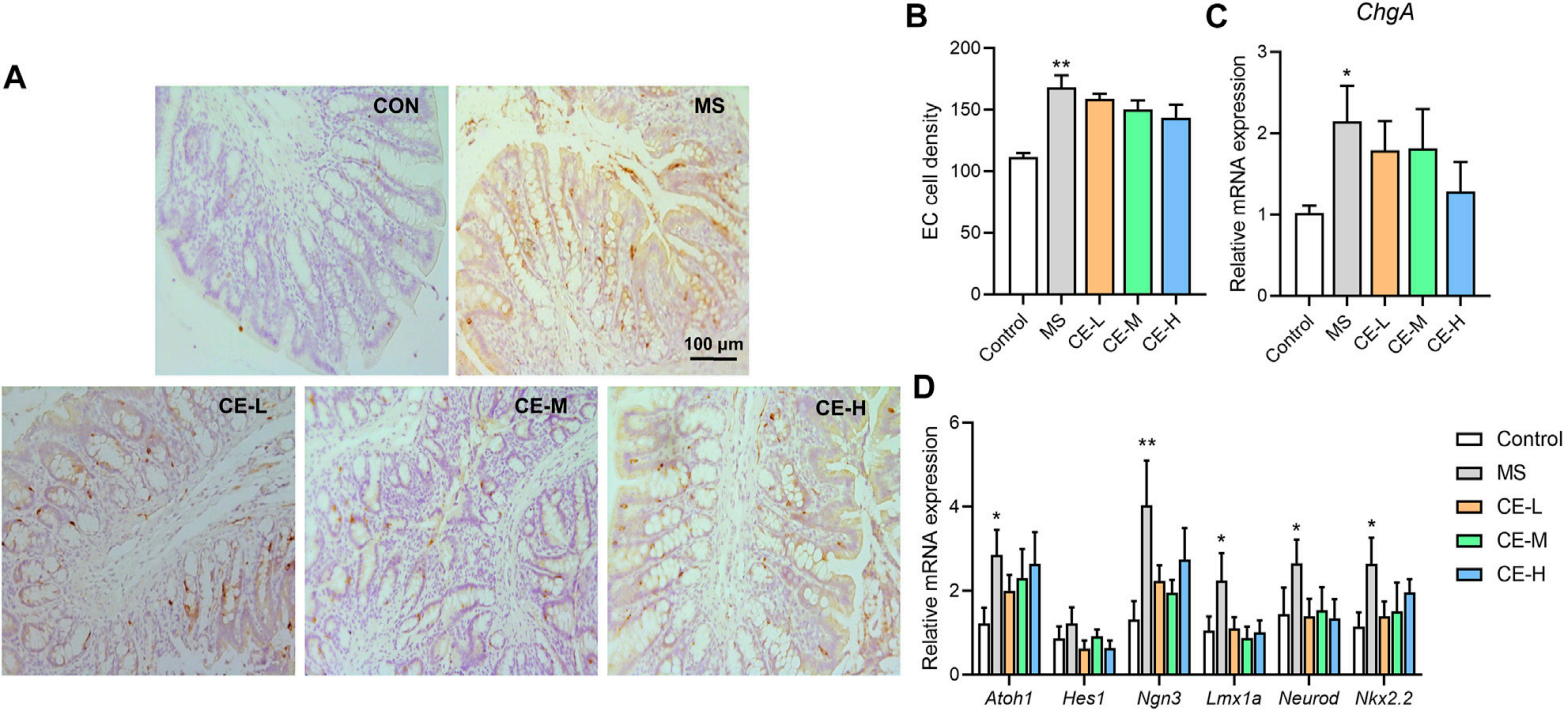
CE has no significant impact on colonic enterochromaffin cell (EC) differentiation in MS rats **(A,B)** Histological images and calculated results of ChgA^+^ cells in the colonic tissues of rats (n = 5/group); **(C,D)** Relative expression of colonic ChgA and genes related to EC cell differentiation (n = 6/group). One-way ANOVA analysis is used for statistics and significance is expressed as *, *p* < .05; **, *p* < .01 compared with the control group.

Given our previous findings that elevated enteric 5-HT levels in MS rats result from increased intestinal stem cell differentiation into enterochromaffin (EC) cells (Wong et al., 2019), it was hypothesized that CE-inhibited 5-HT synthesis may impact colonic EC cell differentiation. Chromogranin A (ChgA), a marker of EC cells, was significantly upregulated in the colonic tissues of MS rats at both the gene and protein level, suggesting that MS stimulates colonic EC cell differentiation. However, CE had no effect on ChgA^+^ EC cell density or colonic ChgA gene expression in MS models (Figures 4A–C). Several regulators involved in EC differentiation were also assessed at the mRNA level. While MS models had increased *Atoh1*, *Ngn3*, *Lmx1a*, *Neurod,* and *Nkx2.2* expression, indicating a higher level of EC differentiation, CE had no effect on the mRNA expression of these differentiation-related proteins (Figure 4D). These findings suggest that CE is unlikely to act on colonic 5-HT synthesis by impacting EC cell differentiation.

### Cinnamon extract and its bioactive components suppress Tph1 transcriptional activity

**FIGURE 5:**
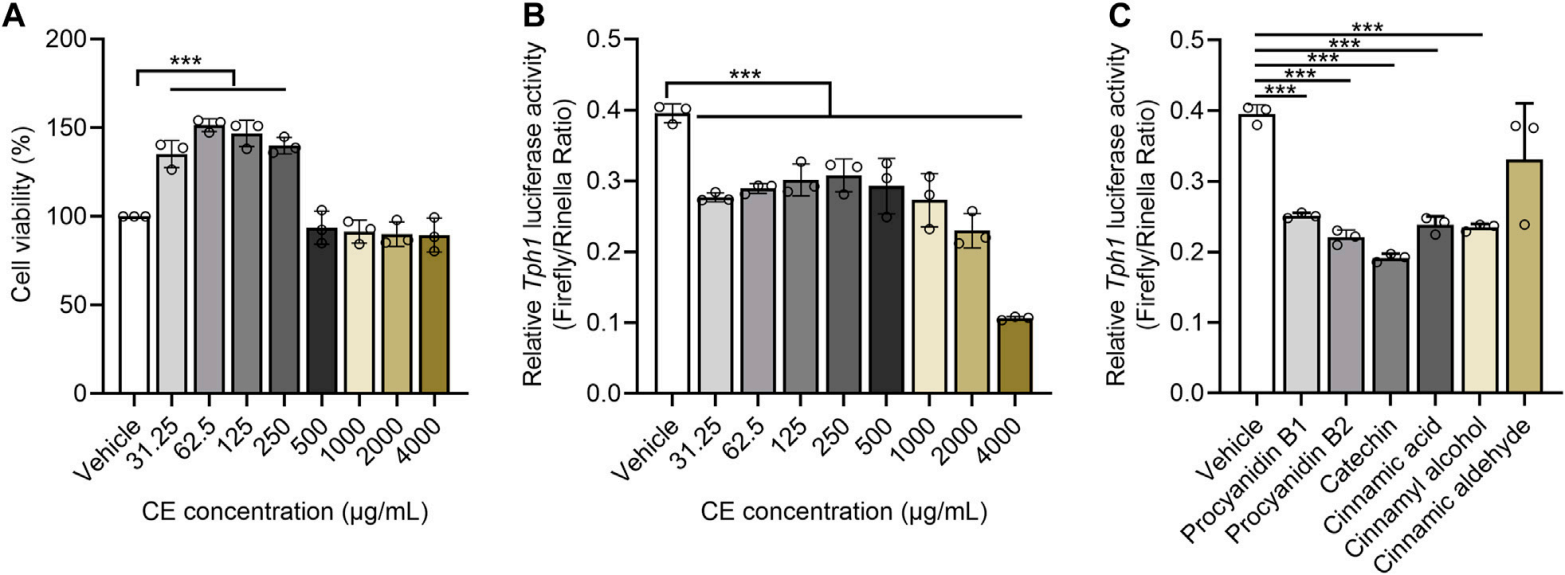
CE and its major components significantly suppress Tph1 transcriptional activity **(A)** The effects of different CE doses on HEK293 cell viability **(B,C)** The impact of CE and its components on Tph1 transcriptional activity resulting from the HEK293-based reporter gene assay. *In vitro* data was tested using three duplicates per group. One way ANOVA analysis was used for statistics and, significance was expressed as ***, *p* < .005 compared with the vehicle group.

CE is thought to directly inhibit Tph1 expression (Noguchi et al., 1973). This was evaluated using an HEK293-based *Tph1* promoter activity assay. After confirming that CE did not have a cytotoxic impact on HEK293 cells (Figure 5A), bioactive gradient dosages of CE (31.25, 62.5, 125, 250, 500, 1000, 2000 and 4000 μg/ml) were used as described in prior *in vitro* studies (Lin et al., 2008; Tung et al., 2008; Kwon et al., 2009). All aqueous CE doses significantly inhibited *Tph1* transcriptional activity, with dose-dependent suppressive action occurring in the 500–4000 μg/ml range (Figure 5B).

UHPLC-QTOF-MS-based phytochemical analysis captured a total of 67 components from CE, five of them were identified as procyanidin B1/B2 (5.45%), Catechin (2.72%), Cinnamic acid (1.44%), Cinnamic aldehyde (1.06%), and Cinnamyl alcohol (0.73%) after matching with the MS/MS fragments of chemical standards (Supplementary Figure S1). Using the IC_50_ values of cinnamon components described *in vitro* (Ruwizhi and Aderibigbe, 2020), catechin, procyanidin B1, procyanidin B2, cinnamic acid, cinnamyl alcohol were shown to individually suppress *Tph1* promoter activity at 50 μΜ; however, cinnamic aldehyde exhibited no effect (Figure 5C). These results suggest that reduced enteric 5-HT production is strongly related to the ability of CE and its chemical ingredients to directly inhibit 5-HT synthetase Tph1 expression.

## Discussion

This study found that CE attenuates bowel symptoms in both chronic stress and chemically induced IBS rat models. The mechanism for the anti-IBS action of CE was shown to involve the suppression of Tph1-dependent colonic 5-HT synthesis. Specifically, aqueous CE and its bioactive components, procyanidin B1, procyanidin B2, catechin, cinnamic acid, and cinnamyl alcohol, significantly inhibited *Tph1* transcriptional activity.

Traditional Chinese Medicine formulas contained cinnamon as an important herbal constitute, such as Gui-Fu-Li-Zhong Decoction and Chai-Hu-Gui-Zhi-Gan-Jiang Decoction, are widely applied to treat IBS, showing significant relief on global bowel symptoms (Li et al., 2013; Xiao et al., 2015). A trial from Australia reported that a cinnamon quills-containing natural formula can improve general symptoms but not bowel movements in IBS-D patients (Hawrelak and Myers, 2010). Another study revealed that aqueous CE from bark had anti-diarrheal activity in mice by reducing GI motility (Hari Jagannadha Rao, 2012). Despite of lacking direct evidence, existing clinical and preclinical works implicate the potential of CE in IBS therapy.

The current study reports that CE reduced defecation frequency *in vivo.* Findings further showed for the first time that CE can reduce visceral hypersensitivity in two different IBS mouse models. While both CE and Ramosetron were unable to impact fecal consistency, however, we cannot exclude a possible effect of these drugs on enteric secretion. No differences in fecal water content are shown in the MS model (Enqi et al., 2020) suggesting that the IBS model used here may not be the most appropriate model to study enteric secretion dysfunction. Moreover, cinnamon was previously reported to decrease intestinal permeability but increase tight junction protein expression *in vitro*, possibly by suppressing phosphorylation NF-κB (Kim and Kim, 2019). The above data indicate that CE has a protective effect on gut dysmotility, visceral hypersensitivity, and restoration of epithelial barrier function.

The dysfunction of peripheral neurotransmitters is considered a pathogenic factor in IBS (Gros et al., 2021). We showed for the first time that CE inhibits IBS by restoring neurotransmitter function. High histamine and 5-HT levels in the colonic tissue and serum of IBS rats suggest that they contribute to bowel motor and sensory dysfunction. While histamine is primarily produced by mast cells and is known to stimulate GI inflammation and cause abdominal pain, 5-HT is synthesized by EC cells and mediates GI secretion, peristalsis, absorption, and visceral sensation (Gershon and Tack, 2007; Fabisiak et al., 2017). CE was shown to suppress both neurotransmitters in the colon but only inhibit 5-HT in the serum, suggesting that the serotonergic system may be more sensitive to CE than the histaminergic system. The ability of high CE doses to attenuate serum acetylcholine levels also suggests that it plays a role in modulating peripheral acetylcholinergic signaling.

Excess 5-HT bioavailability in colonic tissues and serum is thought to be attributed in part to its over-synthesis and/or increased secretion in the colonic mucosa (Fu et al., 2019). Most gut 5-HT is synthesized and stored in EC cells that act as transducers of luminal stimuli (Spiller, 2011). Our previous study found the expansion of intestinal stem cell (ISC) and their differentiation toward secretory lineages resulted in colonic EC hyperplasia and increased 5-HT production in the MS model (Wong et al., 2019). In the current study, CE was able to attenuate elevated 5-HT levels consistently shown in circulation and in the colonic tissue of MS rats and reduce expression of Tph1 in the colon. These findings suggest that CE ameliorates IBS-D in large part by targeting 5-HT synthesis. Moreover, the slight increase in *Sert* gene expression in MS rats and reduced expression in CE-treated model rats suggest that 5-HT transport is not a direct target for CE but may instead be an adaptive response to changes in 5-HT synthesis (Bian et al., 2010).

Unchanged colonic EC cell counts and ChgA gene expression in CE-treated models suggest that CE was unlikely to inhibit colonic 5-HT synthesis by preventing ISC differentiation toward EC cells. Instead, CE may exert its effects by directly inhibiting the expression of 5-HT synthase. Importantly, a possible effect of CE on TPH2, another isoform of 5-HT synthase in the colon, cannot be excluded. While TPH1 and TPH2 are both responsible for 5-HT synthesis, TPH1-generated 5-HT accounts for 90% of total gut 5-HT production while TPH2 expressed by neurons accounts for the remaining 10% (Terry and Margolis, 2017). These two TPH isoforms also have different impacts on GI physiology. While TPH1 mediates colon morphology, and motor and sensory functions, TPH2 regulates GI motility (Gershon, 2013). Thus, the current study focused on TPH1 because it plays a greater role in 5-HT production and GI physiology than TPH2.

The *in vitro* results described here revealed that cinnamic acid, catechin, procyanidin B1/2, and cinnamyl alcohol had a direct suppressive effect on *Tph1* transcription but cinnamic aldehyde did not. While cinnamic acid, catechin, and procyanidin B1/2 are water soluble, cinnamyl alcohol is only slightly soluble and cinnamic aldehyde is miscible in oils. Thus, the inhibitory effect of CE on TPH1 expression is attributed to those components with water solubility. Cinnamic aldehyde, which is predominantly found in the oil of cinnamon bark and shown to act as a TRPA1 agonist and stimulate the release of 5-HT release (Nozawa et al., 2009; Lieder et al., 2020), had no impact on Tph1 transcription in the current study. This suggests that different CE preparations may have varied impacts on GI function, with the aqueous extract showing the highest efficacy against IBS-D.

Moreover, cinnamaldehyde and cinnamic acid have been reported to have synergistic antibacterial effects and combinations of trans-cinnamaldehyde with p-cymene, cinnamyl alcohol, or cinnamic acid exhibit synergistic activity in anti-inflammation (Schink et al., 2018; Huang et al., 2021). It suggests that CE-derived compounds might have synergistic anti-IBS impacts. Also, we cannot rule out there might be other CE-derived bioactive substances in addition to the five compounds mentioned above, owning to a limitation in the phytochemical identification of the aqueous extracts.

IBS is also known to be driven by stress *via* the gut-brain axis (Drossman, 2016), the dysregulated connection between gut and brain is a critical pathogenic factor. We suppose that CE could improve bowel dysfunctions by modulating the gut-brain axis in addition to 5-HT metabolism. CE and volatile components, e.g., eugenol and cinnamaldehyde, have neuroprotective functions, with significant impacts on restoring neuronal loss, suppressing microglial activation, and minimizing stress response by alleviating Hypothalamic-Pituitary-Adrenal (HPA) axis activation (Saxena and Saxena, 2012; Ho et al., 2013; Qubty et al., 2021). Another study revealed that intraluminal 5-HT could activate vagal primary afferent neurons (Zhu et al., 2001), which may thus affect brain signals. It remains need to be further investigated that whether and how CE affects the gut-brain axis.

## Conclusion

This study found that aqueous CE effectively attenuated bowel symptoms, particularly visceral hyperalgesia, in IBS rat models. Results indicated that CE directly suppressed colonic 5-HT synthetase Tph1 expression *in vivo* and, along with its components, catechin, procyanidin B1, procyanidin B2, cinnamic acid and cinnamyl alcohol, significantly inhibited Tph1 transcription *in vitro*. These findings illustrate that aqueous CE effectively ameliorates bowel symptoms by inhibiting colonic Tph1 expression, thereby regulating 5-HT synthesis. This study provides scientific evidence for the use of spices such as cinnamon as folk medicine treatment for IBS.

## Data Availability

The original contributions presented in the study are included in the article/Supplementary Material, further inquiries can be directed to the corresponding authors.
