# Supplementary material for: Aqueous cinnamon extract ameliorates bowel dysfunction and enteric 5-HT synthesis in IBS rats
Source: Front Pharmacol. 2023 Jan 9;13:1010484. doi: 10.3389/fphar.2022.1010484 (PMC9868158; doi:10.3389/fphar.2022.1010484)
Supplement: Supplementary file 1 [file DataSheet1.pdf]

Supplementary materials

Aqueous cinnamon extract ameliorates bowel dysfunction and enteric 5-HT synthesis in IBS rats

Lijuan Yu[1,2#], Chunhua Huang[1#], Wei Yang[1#], Zhenxing Ren[3], Lifeng Li[1], Huiyuan Cheng[1], Chengyuan Lin[1], Lixiang Zhai[1], Ziwan Ning[1], Hoileong Xavier Wong[1], Quanbin Han[1], Wei Jia[1,3], Zhaoxiang Bian[1*], Ling Zhao[1,4*]

1. School of Chinese Medicine, Hong Kong Baptist University, Hong Kong SAR, China
2. College of Basic Medicine, Guangzhou University of Chinese Medicine, Guangzhou, China
3. Center for Translational Medicine, Shanghai Jiao Tong University Affiliated Sixth People's Hospital, Shanghai, China
4. Academy of Integrative Medicine, Shanghai University of Traditional Chinese Medicine, Shanghai, China

[#]Authors share co-first authorship

*Corresponding Author

Correspondence:

Zhao-xiang Bian, MD, PhD

E-mail: bzxiang@hkbu.edu.hk

Ling Zhao, PhD

E-mail: zhangda0525@163.com

This file includes:

Fig S1. Representative chromatograms and mass spectrographs of [M+H] ion masses from the cinnamon extract by UHPLC-QTOF-MS.

Table S1. Primer sequences for qPCR analyses

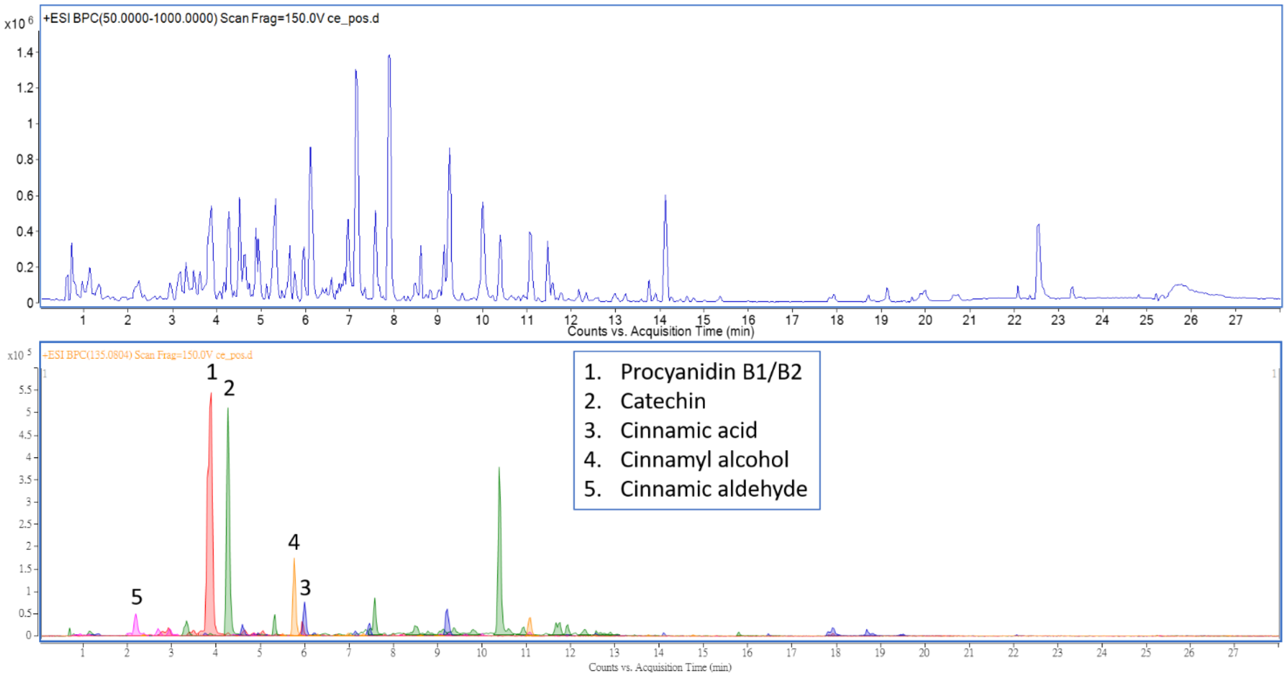

Fig. S1. Representative chromatograms and mass spectrographs of [M+H] ion masses from the cinnamon extract by UHPLC-QTOF-MS. (A) The BPC chart and (B) The EIC chart.

Table S1. Primer sequences for qPCR analyses

**Origins**	**Targeted genes**	**Sequences (5'→3')**
Rat	Mao-A	F: GCCTTCCTGACTTAATCAC
	Mao-A	R: CCTCGATCCCAGTCCTGCC
	Mao-B	F: GCACTGTGGTTCCTGTGGTAT
	Mao-B	R: GCACTGTGGTTCCTGTGGTAT
	TPH1	F: TGCGACATCAACCGAGAACAGC
	TPH1	R: CGGGCGAGTCCACAGAGAGG
	SERT	F: CCACCTTCCCATACATTGT
	SERT-	R: CTGTCTCCAAGAGTTTCTGC
	ChgA	F: ATCACCGCCACTGCCACCACCA
	ChgA	R: CACCTTAGTGTCCCCTTTTGTCATAGGGCT
	Atoh1	F: AACACCGAGTGTGTCTAGCG
	Atoh1	R: GTTTCTTCAAGGCGGCACAG
	Nkx2.2-	F: CGAGTCACCGGACAATGACA
	Nkx2.2	R: GCTTCGATCCTGGCATCCAT
	Lmx1a	F: CCTTGGCTGAACTCTACCCTC
	Lmx1a	R: TATCCGAGATGACCCGCTGA
	Hes1	F: GCCAGTGTCAACACGACACC
	Hes1	R: GCGGTACTTCCCCAACACG
	Ngn3	F: GCAGAGCAGATAAAGCGTGC
	Ngn3	R: TCGCCTGGAGTAAATTGCGT

	NeuroD	F: CTTCCCGGTGCATCCCTACTCCTACC
	NeuroD	R: AGGAAGGGCTGGTGCAATCAGTTAGG
